# Reactive Infectious Mucocutaneous Eruption (RIME) Associated With Rhinovirus and HHV-7 in a 14-Year-Old Boy

**DOI:** 10.1155/crpe/4512323

**Published:** 2025-11-20

**Authors:** Giulia Burati, Fortunato Cassalia, Anna Bolzon, Guido Mioso, Francesca Caroppo, Anna Belloni Fortina

**Affiliations:** ^1^Department of Women and Children's Health, Pediatric Dermatology Regional Center, University of Padua, Padua, Italy; ^2^Department of Medicine (DIMED), Dermatology Unit, University of Padua, Padua, Italy; ^3^Department of Women's and Children's Health, European Reference Network for Rare Skin Disorders-ERN Skin, University of Padua, Padua, Italy

**Keywords:** case report, HHV-7, reactive infectious mucocutaneous eruption, rhinovirus, RIME

## Abstract

Reactive infectious mucocutaneous eruption (RIME) is a recently identified nosological entity that distinguishes certain mucocutaneous reactions caused by bacterial or viral infections from Stevens–Johnson syndrome (SJS). We present the case of a 14-year-old boy who developed mucositis with minimal skin involvement associated with positivity for rhinovirus and HHV-7, which is consistent with a diagnosis of RIME.

**Summary**

• Reactive infectious mucocutaneous eruption (RIME) is a clinical entity distinct from Stevens–Johnson syndrome (SJS), often triggered by *Mycoplasma pneumoniae*.
• It typically presents in children and adolescents with prominent mucosal involvement and limited or absent skin lesions.
• While Mycoplasma is the most recognized cause, other infectious agents such as influenza, enteroviruses, and SARS-CoV-2 have also been implicated.
• This is the first documented pediatric case of RIME potentially associated with HHV-7, either as a primary trigger or as a coreactivating agent.

## 1. Introduction

SJS and toxic epidermal necrolysis (TEN) are severe mucocutaneous disorders that belong to the same pathological spectrum, characterized by extensive epidermal necrosis and skin detachment, typically accompanied by mucosal involvement in 90% of cases [1]. Even if the majority of SJS/TEN cases are associated to drugs such as sulphonamides, nonsteroidal anti-inflammatory drugs, and anticonvulsants, in several cases, the etiology remains unknown [2].

Infectious agents can contribute to the development of SJS/TEN, with Mycoplasma infections identified in approximately 30% of cases [3]. In this context, in 2015, Canavan et al. coined the term “*M. pneumoniae*–induced rash and mucositis” (MIRM) to describe a subset of reactive mucositis with limited skin involvement associated with Mycoplasma infection [4]. Subsequently, Miller et al. introduced the broader concept of “RIME”, encompassing mucocutaneous reactions triggered by various bacterial or viral pathogens, including those such as MIRM [5]. The literature on this topic is recent and continuously evolving.

We report the case of a 14-year-old boy with mucocutaneous manifestations consistent with RIME, associated with rhinovirus and HHV-7 infections.

## 2. Case Report

**Figure 1:**
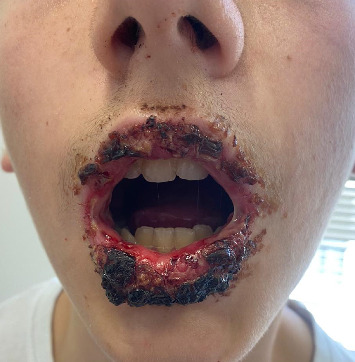
Erosions and hemorrhagic crusts on the lips.

A previously healthy 14-year-old male presented to the pediatric emergency department with a mucocutaneous reaction characterized by widespread stomatitis, with erosions and hemorrhagic crusts on the lips (Figure 1), bilateral follicular blepharoconjunctivitis, and genital involvement, including erythema of the urethral meatus and nonvesicular circular lesions on the scrotum. Five days prior, the patient had developed cough and fever, which were treated with paracetamol. No additional symptoms or medication history were reported. On the following day, he developed mild aphthous stomatitis affecting the labial mucosa and consulted his attending physician, who recommended topical Vitamin E. Two days later, as vesicular lesions spread within the oral cavity and the pain intensified, the patient presented to a peripheral emergency room. There, he was prescribed acyclovir for suspected oral herpes, along with a topical soothing agent. After 5 days of increasing symptoms, he sought care again, where blood tests revealed a C-reactive protein (CRP) of 103.9 mg/L. Hemochromocytometric values showed mild lymphopenia (lymphocytes: 1.1 × 10^9^/L), with other parameters within normal limits. Lymphocyte subsets were not assessed. Based on these findings, he was referred to the emergency department at Padua Hospital.

Upon admission, due to the patient's unremarkable pharmacological history and elevated CRP, extensive microbiological testing was performed. The results revealed positivity for rhinovirus from nasopharyngeal aspirate and HHV-7 from blood polymerase chain reaction (PCR). The nasopharyngeal aspirate was negative for *M. pneumoniae*, *Chlamydia pneumoniae*, adenovirus, bocavirus, SARS-CoV-2, enterovirus, Influenza A/B, RSV, and parainfluenza viruses. Blood PCRs were negative for Parvovirus B19, CMV, EBV, HHV-6, HHV-8, HSV, Mycoplasma, and Rickettsia, and cultures as well as swabs from the urethra and conjunctiva, were also negative. A chest X-ray was unremarkable.

The patient was hospitalized for pain relief therapy and intravenous hydration due to severe oral, ocular, and genital discomfort, along with difficulty eating and dysuria. Dermatological management included topical Vitamin E for oral lesions and a combination of Vitamin E and mometasone furoate 0.1% for genital lesions. Ophthalmological care included eyelid hygiene, artificial tears, and netilmicin-dexamethasone eye drops.

**Figure 2:**
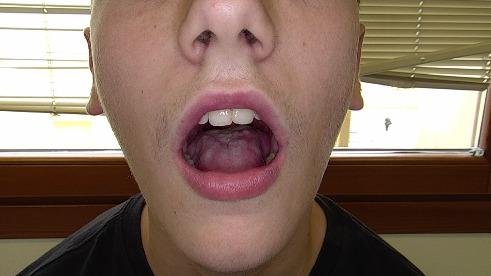
Clinical presentation 6 weeks after therapy.

Within 10 days, the patient showed significant clinical improvement, including resolution of oral lesions and full resumption of oral intake. Six weeks later, follow-up revealed complete resolution of the mucositis, genital lesions, and ocular symptoms (Figure 2).

## 3. Discussion

The clinical presentation of this case aligns with the characteristics of RIME. However, the timing of presentation and the evidence of both rhinovirus and HHV7 makes difficult to address etiological defined agent as the primary cause.

RIME predominantly affects young males and often presents with nonspecific prodromal symptoms such as fever, malaise, and cough. It is characterized by mucosal involvement and ocular involvement in most cases. Genital involvement is often, but not always, reported. Skin involvement is less common, with approximately one-third of patients having no skin manifestations [6].

Although *M. pneumoniae* is the most common pathogen associated with RIME, other bacterial and viral agents, including *C. pneumoniae*, Influenza B, enterovirus, adenovirus, human metapneumovirus, and parainfluenza virus Type 2, have also been implicated [7–11]. In contrast, associations with infections such as VZV, Hepatitis A, EBV, CMV, and HHV-6 are less commonly linked to RIME [5–7]. Recently, several cases of RIME have been associated with SARS-CoV-2 infection [8, 9]. To date, no cases of pediatric RIME related to HHV-7 have been reported. Conversely, rhinovirus has been implicated in pediatric cases of RIME [10].

While establishing a definitive primary trigger in the setting of a dual infection is challenging, in our case, the clinical timeline and existing literature suggest that rhinovirus was the likely initiating pathogen, with HHV-7 potentially acting as a synergistic contributor.

In terms of management, in our case, systemic corticosteroids and immunomodulatory therapies were not administered. Antibiotic treatment was also unnecessary, as no bacterial etiology was identified, and there was no evidence of systemic bacterial infection. The patient was initially treated with acyclovir; however, it was discontinued following negative microbiological testing for HSV. Our therapeutic efforts were focused on symptomatic relief and topical treatment until complete clinical resolution.

A growing body of evidence shows that RIME can be precipitated by a wide spectrum of respiratory pathogens beyond *M. pneumoniae*. Recent reports describe recurrent episodes triggered by SARS-CoV-2 and Influenza A in adolescents [6], sequential flares associated with *M. pneumoniae*, SARS-CoV-2, and rhinovirus in an adult male [9], and a series of cases linked to multiple bacteria and viruses, including rhinovirus and HHV-7, which often cocirculated in the same patient [10].

These observations support the likelihood that, in our patient, rhinovirus acted as the primary trigger, while a concurrent HHV-7 infection contributed synergistically. Together, they appear to have induced immunological cross-talk that amplified T-cell activation and ultimately altered the mucocutaneous response.

This case highlights the need for clinicians to maintain a high index of suspicion for RIME in pediatric patients presenting with mucocutaneous symptoms, particularly due to its overlapping clinical features with simultaneous viral infections. However, given the ubiquity of HHV-7 and its frequent reactivation during systemic illness, we cannot exclude the possibility that its detection represented an incidental finding rather than a direct pathogenic trigger.

## Nomenclature

SJS: Stevens–Johnson syndrome
TEN: Toxic epidermal necrosis
MIRM: *Mycoplasma pneumoniae*–induced rash and mucositis
RIME: Reactive infectious mucocutaneous eruption
